# Reply to ‘Physical limitations on broadband invisibility based on fast-light media’

**DOI:** 10.1038/s41467-021-22974-8

**Published:** 2021-05-24

**Authors:** K. L. Tsakmakidis, O. Reshef, E. Almpanis, G. P. Zouros, E. Mohammadi, D. Saadat, F. Sohrabi, N. Fahimi-Kashani, D. Etezadi, R. W. Boyd, H. Altug

**Affiliations:** 1 Section of Condensed Matter Physics, Department of Physics, National and Kapodistrian University of Athens, Panepistimioupolis, Athens, Greece; 2 Department of Physics and School of Engineering and Computer Science, University of Ottawa, Ottawa, ON Canada; 3 Bioengineering Department, EPFL – École Polytechnique Fédérale de Lausanne, Lausanne, Switzerland; 4 Department of Applied Physics and Institute for Photonic Integration, Eindhoven University of Technology, Eindhoven, Netherlands; 5 Department of Mechanical Engineering, University of Massachusetts Lowell, 1 University Avenue, Lowell, MA USA

**Keywords:** Metamaterials, Optical physics

**Replying to** Abdelrahman et al. *Nature Communications* 10.1038/s41467-021-22972-w 2021

We appreciate the study by Abdelrahman et al.^1^ on whether fast-light cloaks can provide broadband invisibility. Such discussions are, in general, helpful as they can elucidate subtle issues. We are therefore pleased to herein provide further results that clarify and corroborate the findings of our original work^2^. In particular, in the following we show, using a concrete example, that broadband, stable, causal, fast-light aided invisibility is indeed possible for electrically-small objects (even when only gain media are used as a fast-light cloak), and we explain how and where our disagreement with Abdelrahman et al. arises.

Abdelrahman et al. reason that the material model we used as a cloak in ref. ^2^ is unphysical because it has, as they argue, a refractive index Re{*n*} < 1 for frequencies *ω* → ∞. However, we would like to note that this only occurs because Abdelrahman et al. extend outside the visible band (‘targeted’ for invisibility in ref. ^2^) the fitting parameters that we had used for the refractive index of the cloaking material in the frequency-domain calculations of ref. ^2^. The analytic expressions for those frequency-domain calculations were provided in the Supplementary Information of ref. ^2^. Our cloaking material was merely a broadband refractive-index-near-zero (RINZ) material^3^, i.e., it has 0 < Re{*n*} < 1 in the visible band. How such media can be realized, not necessarily using only gain materials (in which case there is no stability issue in the first place)^3^, was outlined in some detail in the section entitled ‘Design considerations’ of ref. ^2^.

In general, the spectrum of a medium may exhibit many resonances, and for frequency-domain calculations, such as those in ref. ^2^, one may fit a refractive index function to the spectrum to describe the medium locally, in a specified frequency region—but that fit cannot be used outside the specified frequency region, e.g., all the way to ‘infinite’ frequencies. In the present case, because Abdelrahman et al. unwarrantedly extend and deploy the same RINZ material model (function) for all frequencies, even outside the visible band where it was strictly used, its RINZ behavior (Re{*n*} < 1) survives even for frequencies *ω* → ∞, thereby giving an incorrect impression that our medium is allegedly not causal (Re{*n*} not being equal to unity for *ω* → ∞). Specifically, Abdelrahman et al. inaccurately state that their Eqs. (1) & (2), giving the electric and magnetic susceptibilities *χ*_e_(*ω*) and *χ*_μ_(*ω*) of (an example of the) fitting parameters that we had provided them, were used for frequencies [0, +∞], whereas in fact in ref. ^2^ they were describing the cloaking medium only in the region [0, 1600 THz], fitting accurately its refractive index in the visible band. By contrast, time-domain calculations do require the optical parameters to be defined in the whole frequency interval [0, +∞]. We would like to emphasize here that it is one thing posing the question whether such a RINZ medium can meaningfully be used for providing stable, causal, broadband, fast-light aided invisibility (a legitimate scientific question, addressed below), and another, very different, issue inadvertently mishandling provided material parameters, thereby creating an unfitting impression that the material model used in ref. ^2^ was allegedly acausal.

We stress that in ref. ^2^ we repeatedly specified that, owing to causality, it must be that *n* → 1 as *f* → ∞, as was, e.g., explained both in the Supplementary Information of ref. ^2^ as well as in Fig. 1b of ref. ^2^ In particular, the entire theory developed in ref. ^2^ rested on precisely that assumption, namely that the susceptibilities should → 0 as *ω* → ∞ (equivalently, *ε* → *ε*_0_ as *ω* → ∞). Indeed, detailing the theory of the main paper, in its Suppl. Inform. we explicitly mention [right after Eq. (S13)] that: “…The crucial point, now, is that on the semicircle H_R_^–^ it is |ω| = R → ∞, hence the wavevector k will equal its free-space value, k_0_ = ω/c, because $\mathop{\mathrm{lim}}\limits_{{\omega }\to \infty }{\varepsilon }({\omega })={{\varepsilon }}_{0}.$ …”, with many similar points and statements found throughout ref. ^2^. Moreover, the same point was also, even pictorially, shown in Fig. 1b of ref. ^2^. The front discontinuity, i.e., the part of the incident waveform corresponding to *ω* → ∞, always travels with the speed of light in vacuum, *c*, as shown in those figures, exactly because it must always be (owing to causality) that *n* → 1 as *f* → ∞. Therefore, the statements made by Abdelrahman et al. throughout, at least, the first half of ref. ^1^, where, e.g., they emphasize the notions of causality, front velocity, information velocity, and so forth, implying that those points and notions were not first made and fully taken into account in ref. ^2^, are unfortunately rather unwarranted criticisms.

Having said these, it is nonetheless still legitimate to inquire whether such a RINZ medium (in the visible band) giving rise to stable, broadband, ‘true’ (i.e., interferometrically too) invisibility in the visible band could also be described by a single causal *n*(*ω*) function for all frequencies, i.e., without that function being only a fit of the material response in the visible band, so that *n*(*ω*) could then be used even for direct time-domain simulations, such as e.g., those performed by Abdelrahman et al. in ref. ^1^.

Such a feat is of course possible, i.e., as reported in ref. ^2^ (e.g., section ‘Design considerations’), one may always identify a material simultaneously fulfilling all of the above requirements – with the fundamental reason for this being the fact that the objects considered in ref. ^2^ were electrically small, that is, well within size-bandwidths limits reported previously for invisibility. Indeed, in Fig. 1 below, we present an example (‘alternative cloak’) of a gain doublet medium (there was no need for a third gain resonance at higher frequencies as used by Abdelrahman et al.) which is, simultaneously, causal [i.e., no *n*(*ω*) fit, but a single *n*(*ω*) function for all frequencies, with the property that *ε* → *ε*_0_ & *μ* → *μ*_0_ as *ω* → ∞, or, equivalently, *n* → 1 as *ω* → ∞], leads to the same (in fact, slightly better) scattering cross section (SCS) reduction performance over the entire visible band compared with Ref. ^2^, is characterized by superluminal or negative group velocities (‘fast light’) in the visible band, and gives rise to stable scattering poles up to extremely high frequencies of around 16000 THz (free-space wavelength of less than 20 nm). The permittivity and permeability of the cloaking layer are, specifically, given by:

1 $$\varepsilon =1+\frac{{f}_{\varepsilon 1}{\omega }_{\varepsilon p1}^{2}}{{\omega }_{\varepsilon r1}^{2}-{\omega }^{2}+i\omega {\gamma }_{\varepsilon 1}}+\frac{{f}_{\varepsilon 2}{\omega }_{\varepsilon p2}^{2}}{{\omega }_{\varepsilon r2}^{2}-{\omega }^{2}+i\omega {\gamma }_{\varepsilon 2}},$$

2 $$\mu =1+\frac{{f}_{\mu 1}{\omega }_{\mu p1}^{2}}{{\omega }_{\mu r1}^{2}-{\omega }^{2}+i\omega {\gamma }_{\mu 1}}+\frac{{f}_{\mu 2}{\omega }_{\mu p2}^{2}}{{\omega }_{\mu r2}^{2}-{\omega }^{2}+i\omega {\gamma }_{\mu 2}},$$

where *f*_*ε*1_ = *f*_*ε*2_ = *f*_*μ*1_ = *f*_*μ*2_ = −1, *ω*_*εr*1_ = *ω*_*μr*1_ = 0.05∙2*π*∙500 10^12^ Hz, ${\omega }_{\varepsilon p1}=\sqrt{0.66}\cdot {\omega }_{\varepsilon r1}$, *ω*_*εr*2_ = *ω*_*μr*2_ = 3∙2*π*∙500∙10^12^ Hz, ${\omega }_{\varepsilon p2}=\sqrt{0.67}\cdot {\omega }_{\varepsilon r2}$ Hz, *γ*_*ε*1_ = *γ*_*ε*2_ = 0.06∙2*π*∙500 10^12^ Hz, ${\omega }_{\mu p1}=\sqrt{0.665}\cdot {\omega }_{\mu r1}$, ${\omega }_{\mu p2}=\sqrt{0.675}\cdot {\omega }_{\mu r2},$ *γ*_*μ*1_ = *γ*_*μ*2_ = 0.01∙2π∙500∙10^12^ Hz.

**Fig. 1:**
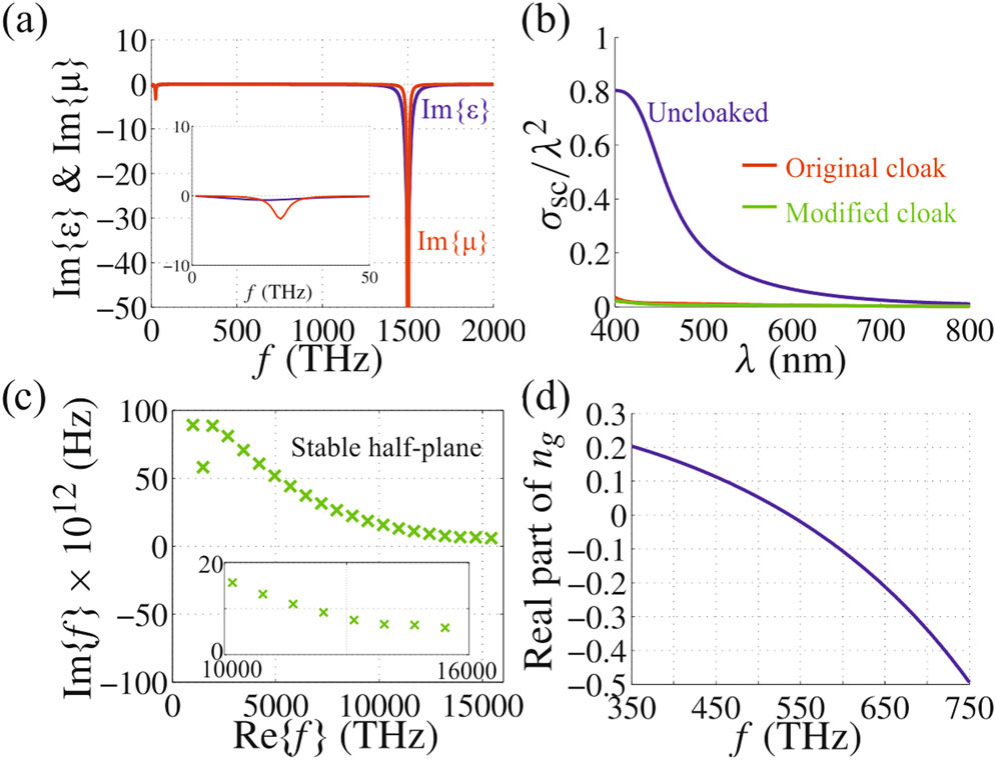
Broadband, stable, causal, fast-light aided invisibility cloaking of electrically small objects. **a** Imaginary parts of the (relative) permittivity, *ε*, and permeability, *μ*, versus frequency of the gain doublet cloaking medium. The inset shows the same graph focused around the first resonance. **b** Scattering-cross-section performance across the visible regime of the cloak of ref. ^2^ (red line) and of the cloak with the parameters of **a** (green line). **c** Poles of the electric dipolar scattering coefficient on the complex plane, for frequencies up to ~16,000 THz. All poles lie on the upper (stable) half-plane, and the last pole that is technically possible to identify reliably^4–6^ is well above zero {Im{*f*_*i*_} ~ 5,84×10^12^}. The inset shows the same graph but focused around the end of the curve. **d** Group refractive index in the cloaking layer of **a**, showing that the group velocity in this medium is superluminal (0 < *n*_*g*_ < 1), or even negative (*n*_*g*_ < 0), in the visible band targeted for invisibility in, both, ref. ^2^ and herein.

The above frequency regime (~15000 THz – 16000 THz) is the one up to which the identification of poles is technically reliable using state-of-the-art routines^4–6^, since beyond it the magnitude of the complex function giving the poles becomes very small and fluctuating (‘noisy’). We note that even in that regime the imaginary part of the last reliably identified pole (15419 + *i*5,843 ΤHz) is large and positive, Im{*f*_*i*_} ~ 5,84 × 10^12^, i.e., we are well within the stable regime (a potential crossing to the unstable plane is not expected, if the trend shown in Fig. 1c remains smoothly decaying, for even much higher frequencies). Furthermore, in that regime the wavelength inside the material is already less than ~5 nm; therefore, the study of the stability of such a structure for higher frequencies becomes a rather ‘theoretical’ exercise, with no discernible practical consequences, as in that case various important damping mechanisms, such as surface roughnesses, Landau damping, material imperfections (inhomogeneities), and losses in the core material (Si) too, become prominent, providing additional loss channels, which not only balance out the gain of the cloak (stabilize the system) but in fact make the structure overall lossy at those extremely high frequencies.

Further, Abdelrahman et al. mention that ref. ^2^ purports to show “arbitrarily broadband invisibility” by quoting only a part of a sentence from ref. ^2^ (“over any desired band”), unfortunately omitting the remaining part of the same sentence, which in its entirety clearly stated that: “Exploiting such media, one may achieve broadband tachyonic cloaking over any desired frequency band, so long as the superluminality condition (average, dispersive, group velocity larger than the speed of light in vacuum, < v_g_> > c) is attained over the desired bandwidth, …”. In other words, the invisibility bandwidth is not ‘arbitrary’ but constrained by the bandwidth over which the deployed fast-light cloaking material exists. Thus, this criticism too by Abdelrahman et al. is, in our opinion, unfortunately unwarranted.

Finally, we note that almost the totality of the discussions by Abdelrahman et al. at the points where they remind about well-established and known properties of fast light, implying that those properties were allegedly not already explained or indeed strictly adhered to in ref. ^2^, regrettably place our work in a rather distorted context. As an example, in the introduction of ref. ^1^. Abdelrahman et al. go to some extent in trying to convince that because of causality the refractive index of a medium must become unity at ‘infinite’ frequencies, as if that very property (reflected in the front velocity, which corresponds to *ω* → ∞, always propagating with the speed of light in vacuum, *c*) was not already explicitly shown by us in Fig. 1b of ref. ^2^, or as if, in detailing the theory of our main paper in its Supplementary Information, we had not explicitly mentioned [right after Eq. (S13)] that *n* → 1 with *ω* → ∞.

In summary, the disagreement with Abdelrahman et al. arises from the fact that in their time-domain calculations they used outside the regime of their validity and definition – disregarding what was mentioned and explained in the Supplementary Information of ref. ^2^, as well as shown in Fig. 1b of the main paper^2^ – the material (fit) model that we had deployed in ref. ^2^. The further results presented here help to clarify and establish that for electrically small objects, such as those studied in ref. ^2^, the answer to the question posed by ref. ^1^ is in the affirmative: It is indeed possible to attain fast-light aided ultrabroadband invisibility using a stable, causal, refractive-index near-zero^3^ cloaking material.

## Data Availability

The data that support the findings of this study are available from the corresponding author upon reasonable request.
